# Supplementary material for: The Effect of Fusarium verticillioides Fumonisins on Fatty Acids, Sphingolipids, and Oxylipins in Maize Germlings
Source: Int J Mol Sci. 2021 Feb 28;22(5):2435. doi: 10.3390/ijms22052435 (PMC7957515; doi:10.3390/ijms22052435)
Supplement: Supplementary file 1 [file ijms-22-02435-s001.pdf]

Table S1. Oxylipin and fatty acid amount monitored by HPLC-MS/MS in maize seedlings mock-infected and infected with *F. verticillioides* wild type and *fum1Δ* mutant.

Fatty acids detected by HPLC-MS/MS

- (white) not significantly reduced
- (light grey) significantly reduced in *fum1Δ*
- (dark grey) significantly reduced in WT

		C16:0		C16:1		C18:0		C18:1		C18:2		C18:3	
2	ctr	21776		12487	b	129530	b	1132296		3959127		74171	b
	wt	17003	ns	20958	b	222090	b	973026	ns	3947774	ns	103800	b
	fum1Δ	14324		30212	a	387503	a	1193441		4980514		158565	a
4	ctr	22108		21085	b	210274	a	954243	a	4287802	a	110046	a
	wt	8717	ns	15165	c	106922	b	807771	b	2896428	c	83374	b
	fum1Δ	13052		24750	a	195177	a	928479	a	3899950	b	125251	a
7	ctr	21386		20244	c	170488	c	704294		3700789	ab	109418	b
	wt	26606	ns	37777	a	641396	a	847156	ns	4192591	a	154513	a
	fum1Δ	13931		28121	b	451686	b	737351		3469195	b	117696	b
14	ctr	20201		22879	c	137796	c	394614	b	3216646	c	153343	b
	wt	148408	ns	103254	a	1283572	a	897410	a	4824629	a	271208	a
	fum1Δ	198317		92377	b	873651	b	717491	a	4034731	b	282916	a

		C22:0		C24:0		C24:1	
2	ctr	4054898		153579		9037	b
	wt	4076607	ns	139799	ns	19329	a
	fum1Δ	5236141		175796		16506	a
4	ctr	7160139	a	149603	a	19323	a
	wt	5388358	b	124116	b	9995	b
	fum1Δ	4529098	b	136701	ab	12357	ab
7	ctr	10458326	a	167660	a	34973	a
	wt	6768957	b	139432	ab	21167	b
	fum1Δ	4713274	b	118615	b	18184	b
14	ctr	14721658	a	179049	a	24611	
	wt	6802796	b	150451	a	30540	ns
	fum1Δ	4823374	b	103551	b	27930	

Oxylipins detected by LC-MS/MS

not significantly reduced
significantly reduced in *fum1Δ*
significantly reduced in WT

		10-HOME		10-HpOME		9-HODE		9-HpODE		13-HODE		13-HpODE	
	ctr	560	b	718		46514	b	4292	c	142582	b	3155	
2	*wt*	681	ab	1150	ns	57253	b	5646	b	144002	b	2045	ns
	fum1Δ	1152	a	1013		107532	a	10734	a	300503	a	2877	
	ctr	768	a	1149		46796		15618		136643	a	10304	
4	*wt*	363	c	1455	ns	36019	ns	8947	ns	106618	b	8608	ns
	fum1Δ	490	b	1008		47528		10142		128113	a	8783	
	ctr	451		1780	b	56172	b	19213		106308	b	37549	a
7	*wt*	615	ns	4073	a	79043	a	24671	ns	126580	ab	16268	c
	fum1Δ	563		2234	ab	70146	ab	22943		131332	b	23301	b
	ctr	479	b	1295		70289	b	21503	b	129644	b	56424	a
14	*wt*	818	ab	3062	ns	133988	a	34016	a	217697	a	20519	b
	fum1Δ	886	a	2336		127458	a	38209	a	211757	a	12010	b

		9,10-DiHOME		12,13-DiHOME		9-HOTrE		13-HOTrE		9-oxoODE		9-oxoOTrE	
	ctr	10102		17442	b	1427	b	10617	b	29084	b	366	b
2	*wt*	20181	ns	19463	b	1447	b	9897	b	37471	ab	584	ab
	fum1Δ	22924		30157	a	2476	a	18014	a	54106	a	921	a
	ctr	25744		19789		3240		35451	a	119730		1374	
4	*wt*	11183	ns	16089	ns	2074	ns	29936	b	65303	ns	1101	ns
	fum1Δ	24567		24018		2242		30288	b	76364		1088	
	ctr	21923	b	36348		1904		20787	b	104416	ab	2052	ab
7	*wt*	64394	a	44324	ns	3518	ns	28723	a	131368	a	2949	a
	fum1Δ	35650	ab	28059		2941		26755	ab	92606	b	1704	b
	ctr	24964	b	32960		1655		19979	c	118871	b	3738	
14	*wt*	64757	a	30003	ns	4579	ns	32816	b	162063	a	3958	ns
	fum1Δ	62755	a	27310		4055		38080	a	166020	a	3274	

Table S2. MRM and SIM conditions for HPLC-MS/MS analysis. a) Sphingolipids; b) Oxylipins; c) Fatty acids.

a) **Sphingolipids**

Compounds characterized in:

maize
fusarium
both

Sphingoid bases MRM conditions

Compound name	precursor ion	Prod ion	Transition	qualifier	Qualifier id	CE (eV)	Fragmentor (V)
d18:2	298	262	$[M+H-2H_2O]^+$	250	$[M+H-H_2O-HCOOH]^+$	20	80
d18:1	300	264	$[M+H-2H_2O]^+$	252	$[M+H-H_2O-HCOOH]^+$	20	80
d18:0	302	266	$[M+H-2H_2O]^+$	284	$[M+H-H_2O]^+$	20	80
t18:0	318	282	$[M+H-2H_2O]^+$	264	$[M+H-H_2O]^+$	22	80

Ceramides MRM conditions

Compound name	precursor ion	Prod ion	Transition	qualifier	Qualifier id	CE (eV)	Fragmentor (V)
d17:1/18:0	552	250	[base-2H2O]+	268	[base-H2O]+	28	140
d18:2/14:1	506	262	[base-2H2O]+	280	[base-H2O]+	20	140
d18:2/16:1	534	262	[base-2H2O]+	280	[base-H2O]+	26	140
d18:2/16:0	536	262	[base-2H2O]+	280	[base-H2O]+	26	140
d18:2/18:1	562	262	[base-2H2O]+	280	[base-H2O]+	24	140
d18:2/22:1	618	262	[base-2H2O]+	280	[base-H2O]+	28	140
d18:1/16:0	538	264	[base-2H2O]+	282	[base-H2O]+	28	140
d18:1/h22:0	638	264	[base-2H2O]+	282	[base-H2O]+	28	140
d18:0/16:0	540	266	[base-2H2O]+	284	[base-H2O]+	26	140

d18:0/18:2	564	266	[base-2H2O]+	284	[base-H2O]+	26	140
d18:0/18:1	566	266	[base-2H2O]+	284	[base-H2O]+	30	140
d18:0/18:0	568	266	[base-2H2O]+	284	[base-H2O]+	26	140
d18:0/h17:0	570	266	[base-2H2O]+	284	[base-H2O]+	26	140
d18:0/20:0	596	266	[base-2H2O]+	284	[base-H2O]+	28	140
d18:0/24:0	652	266	[base-2H2O]+	284	[base-H2O]+	24	140

PhytoCers MRM conditions

Compound name	precursor ion	Prod ion	Transition	qualifier	Qualifier id	CE (eV)	Fragmentor (V)
t18:0/16:0	556	282	[base-2H2O]+	300	[base-H2O]+	24	140
t18:0/h15:0	558	282	[base-2H2O]+	300	[base-H2O]+	26	140
t18:0/18:2	580	282	[base-2H2O]+	300	[base-H2O]+	30	140
t18:0/18:1	582	282	[base-2H2O]+	300	[base-H2O]+	30	140
t18:0/18:0	584	282	[base-2H2O]+	300	[base-H2O]+	32	140
t18:0/19:1	596	282	[base-2H2O]+	300	[base-H2O]+	28	140
t18:0/20:0	612	282	[base-2H2O]+	300	[base-H2O]+	28	140
t18:0/22:0	640	282	[base-2H2O]+	300	[base-H2O]+	26	140
t18:0/23:0	654	282	[base-2H2O]+	300	[base-H2O]+	24	140
t18:0/24:0	668	282	[base-2H2O]+	300	[base-H2O]+	24	140
t18:0/25:0	682	282	[base-2H2O]+	300	[base-H2O]+	28	140
t18/h22:0	656	282	[base-2H2O]+	300	[base-H2O]+	24	140
t18:0/h23:0	670	282	[base-2H2O]+	300	[base-H2O]+	28	140
t18:0/h24:0	684	282	[base-2H2O]+	300	[base-H2O]+	26	140
t18:0/26:0	696	282	[base-2H2O]+	300	[base-H2O]+	30	140
t18:0/h25:0	698	282	[base-2H2O]+	300	[base-H2O]+	26	140

DehydroPhytoCers MRM conditions

Compound name	precursor ion	Prod ion	Transition	qualifier	Qualifier id	CE (eV)	Fragmento r (V)

t18:1/16:0	554	280	[base-2H2O]+	298	[base-H2O]+	28	140
t18:1/h20:0	626	280	[base-2H2O]+	298	[base-H2O]+	30	140
t18:1/h22:0	654	280	[base-2H2O]+	298	[base-H2O]+	26	140
t18:1/h24:0	682	280	[base-2H2O]+	298	[base-H2O]+	28	140
t18:1/h26:0	710	280	[base-2H2O]+	298	[base-H2O]+	28	140

GlcCers MRM conditions

Compound name	precursor ion	Prod ion	Transition	qualifier	Qualifier id	CE (eV)	Fragmentor (V)
Glc d18:2/16:1	696	262	[base-2H2O]+	280	[base-H2O]+	28	140
Glc d18:2/16:0	698	262	[base-2H2O]+	280	[base-H2O]+	28	140
Glc d18:2/18:1	724	262	[base-2H2O]+	280	[base-H2O]+	26	140
Glc d18:2/20:0	754	262	[base-2H2O]+	280	[base-H2O]+	30	140
Glc d18:2/h20:0	770	262	[base-2H2O]+	280	[base-H2O]+	28	140
Glc d18:2/h22:0	798	262	[base-2H2O]+	280	[base-H2O]+	28	140
Glc d18:2/24:1	808	262	[base-2H2O]+	280	[base-H2O]+	30	140
Glc d18:2/25:1	822	262	[base-2H2O]+	280	[base-H2O]+	30	140
Glc d18:2/26:1	836	262	[base-2H2O]+	280	[base-H2O]+	30	140

d19:2 GlcCers MRM conditions

Compound name	precursor ion	Prod ion	Transition	qualifier	Qualifier id	CE (eV)	Fragmentor (V)
Glcd19:2/14:2	680	276	[base-2H2O]+	294	[base-H2O]+	26	140
Glcd19:2/16:1	710	276	[base-2H2O]+	294	[base-H2O]+	26	140
Glcd19:2/18:2	736	276	[base-2H2O]+	294	[base-H2O]+	26	140
Glcd19:2/19:0	754	276	[base-2H2O]+	294	[base-H2O]+	26	140
Glcd19:2/24:1	822	276	[base-2H2O]+	294	[base-H2O]+	26	140

b) Oxylipin MRM condition

Compound name	precursor ion	Prod ion	Polarity	CE (eV)	Fragmentor (V)
7,10 di HOME	313.3	139.1	$[M-H]^-$	34	140
10-HpOME	313.2	251.1	$[M-H]^-$	28	80
9,10-DiHOME	313.2	201.1	$[M-H]^-$	14	140
12,13-DiHOME	313.2	183.1	$[M-H]^-$	18	140
9,10-DiHOME	313.2	171.2	$[M-H]^-$	14	140
10-HpOME	313.2	155.2	$[M-H]^-$	28	80
12,13-DiHOME	313.2	129.2	$[M-H]^-$	18	140
9-HpODE	311.1	185.2	$[M-H]^-$	14	80
13-HpODE	311.1	113.2	$[M-H]^-$	14	80
9-HODEd4	299.2	172.2	$[M-H]^-$	20	140
10-HOME	297.2	171	$[M-H]^-$	35	140
10-HOME	297.2	155	$[M-H]^-$	35	140
13-HODE	295.2	195.2	$[M-H]^-$	20	140
9-HODE	295.2	171.2	$[M-H]^-$	25	140
13-HOTrE	293.2	223.2	$[M-H]^-$	18	140
13-HOTrE	293.2	195.2	$[M-H]^-$	18	140
9-oxoODE	293.2	185.2	$[M-H]^-$	18	140
9-HOTrE	293.2	171.2	$[M-H]^-$	18	140
13-oxoODE	293.2	170.6	$[M-H]^-$	30	80
9-HOTrE	293.2	121.2	$[M-H]^-$	18	140
13-oxoODE	293.2	113.6	$[M-H]^-$	30	80
9-oxoOTrE	291.4	185.2	$[M-H]^-$	18	140

c) Fatty acid SIM conditions

Compound name	SIM ion	Polarity	Fragmentor (V)
16:0	255.2	140	140
16:1	253.2	140	140
18:0	283.2	140	140
18:1	281.2	140	140
18:2	279.2	140	140
18:3	277.2	140	140
22:0	339.2	140	140
24:0	367.4	140	140
24:1	365.4	140	140

Figure S1. A) Total ion chromatogram of ceramides/glucosyl ceramides MRM method. *F. verticillioides* in red, maize in black; B) Total ion chromatogram of PhytoCeramides/ DehydroPhytoCeramides MRM method. *F. verticillioides* in blue, maize in green.

A)

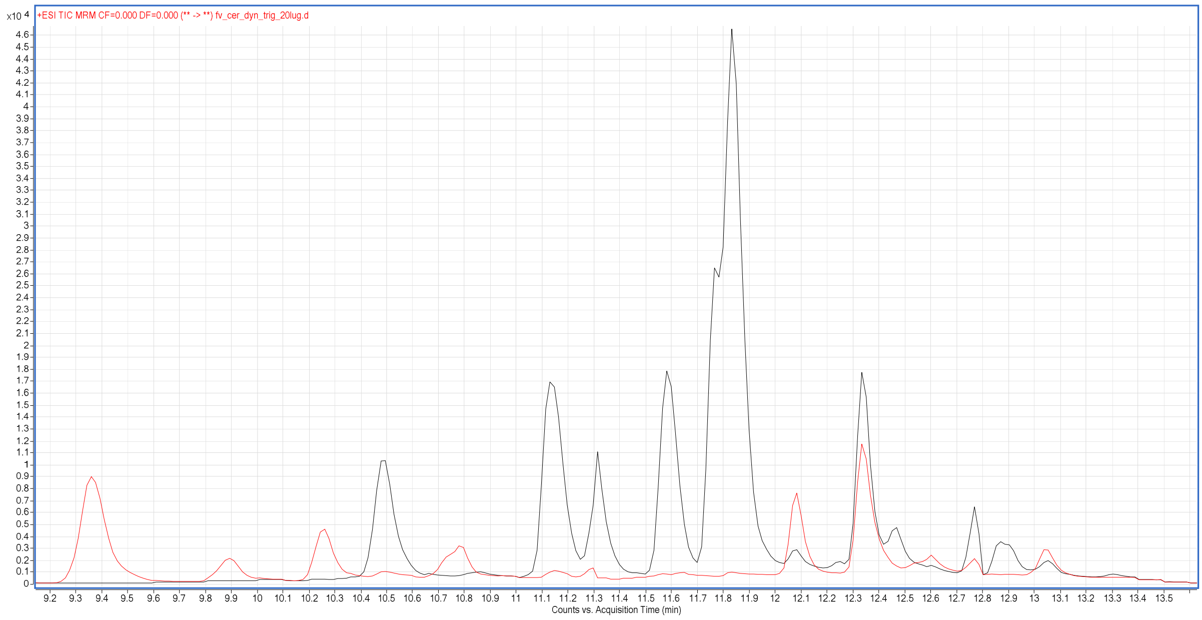

B)

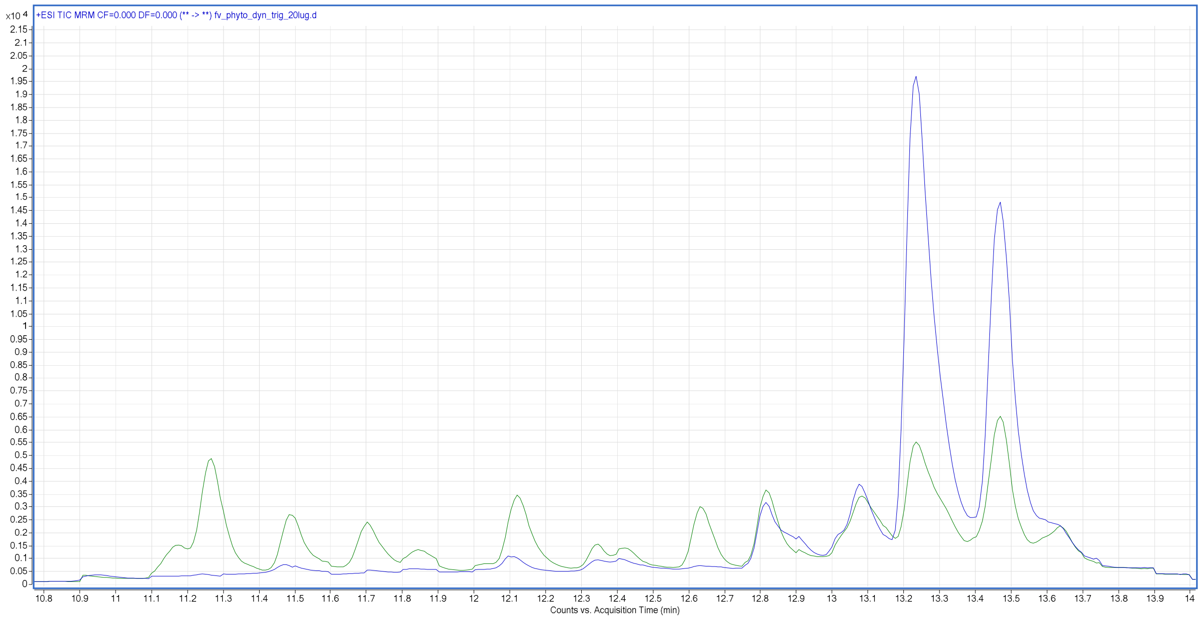

Figure S2. Relative abundance of different sphingolipids. Y-axis: relative abundance of compounds found in maize kernel mock-infected and infected with *F. verticillioides* wild type or *fum1Δ* mutant, measured as normalized peak intensity. X-axis: time of inoculation (2,4,7,14 dai). Box plot represent the distribution of the relative quantity of sphingolipids deriving from three independent replicates, technically repeated in trice. Letters indicate statistically significant differences between infected-maize (wild type or *fum1Δ*) and mock at each time point. Absence of letters indicate that the difference are not statistically significant. Statistically significant differences were evaluated through ANOVA followed by post - hoc Tukey test (*p< 0.05; **p < 0.01; ***p<0.001).

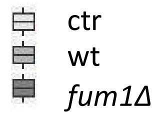

a)

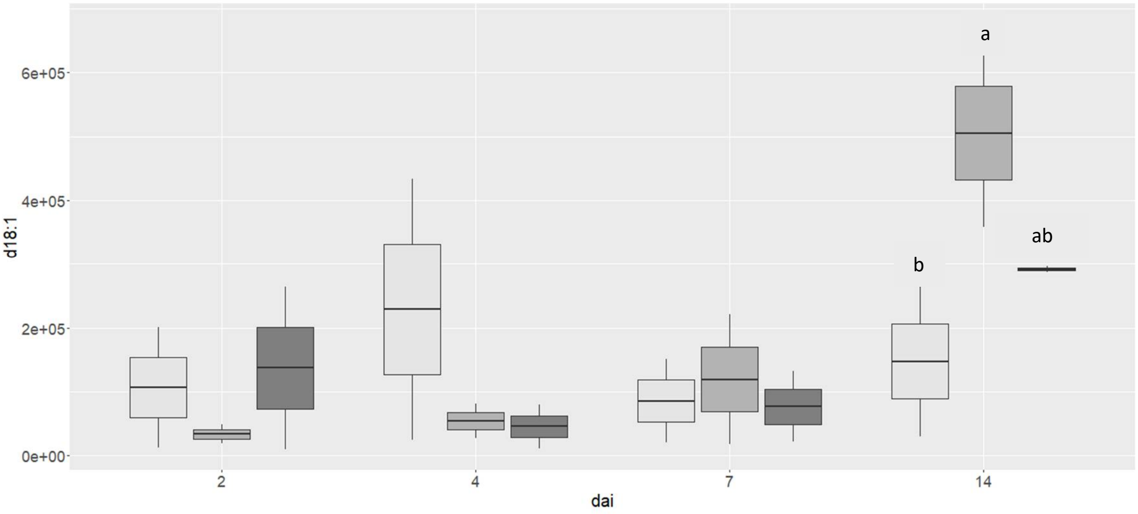

b)

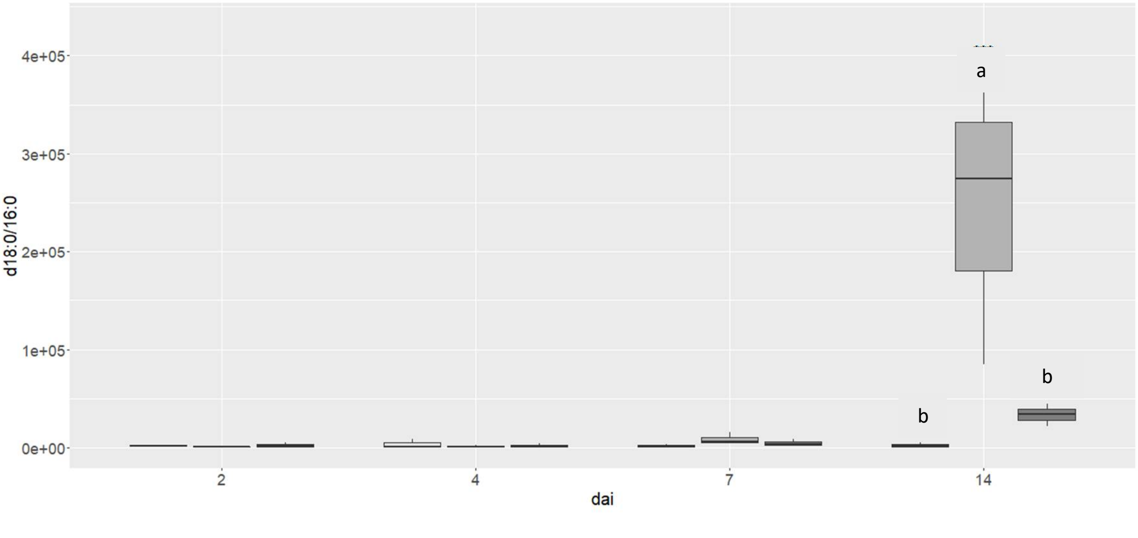

c)

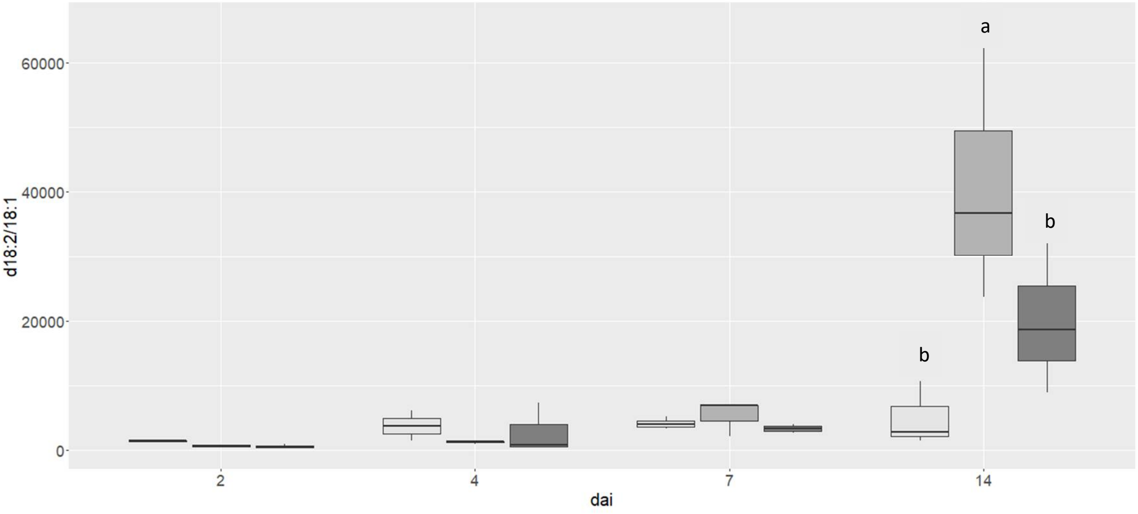

a
b
b
60000
40000
20000
0
d18:2/18:1
2
4
7
14
dai

d)

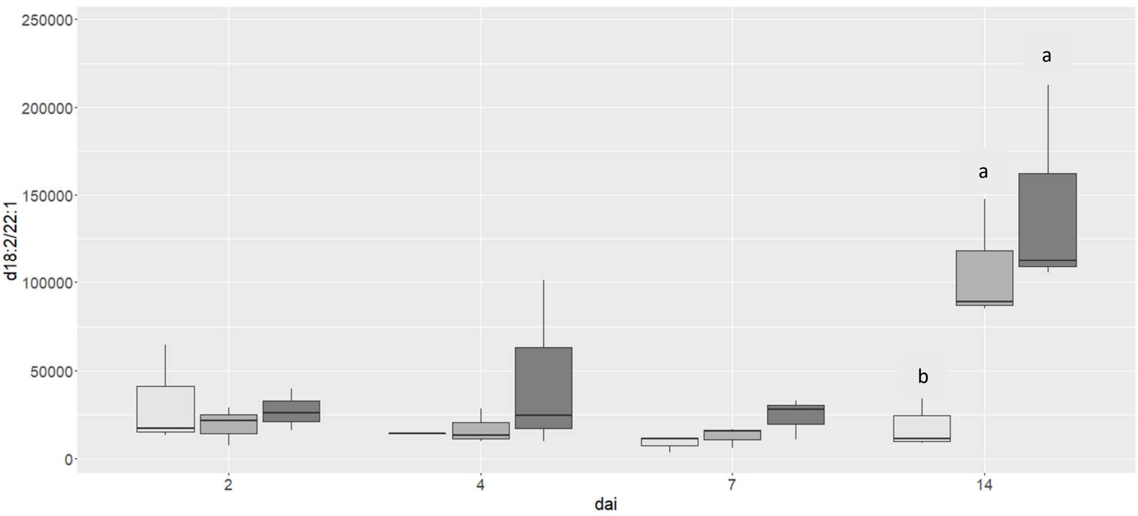

a
a
b
250000
200000
150000
100000
50000
0
d18:2/22:1
2
4
7
14
dai

e)

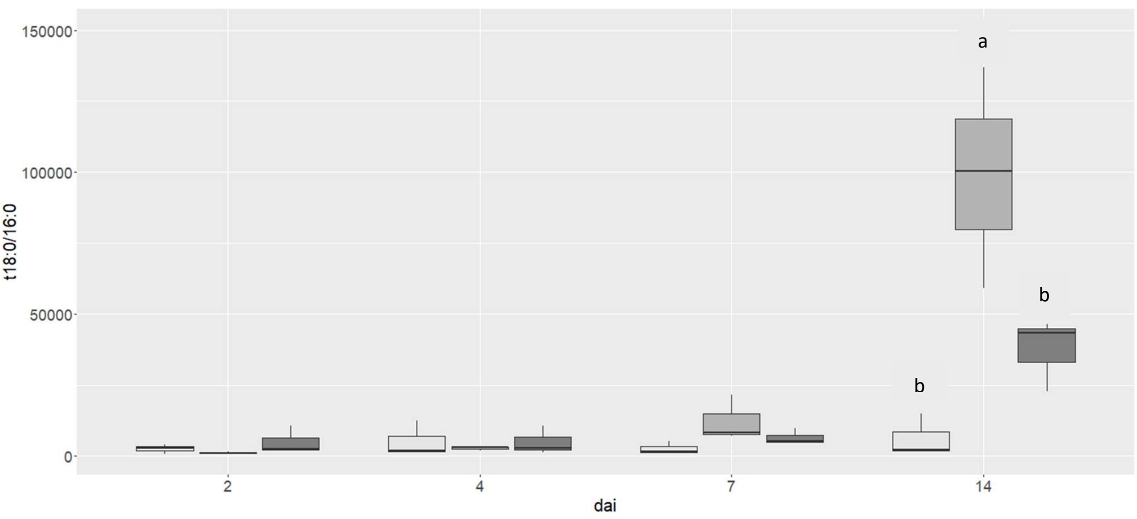

a
b
b
150000
100000
50000
0
t18:0/16:0
2
4
7
14
dai

f)

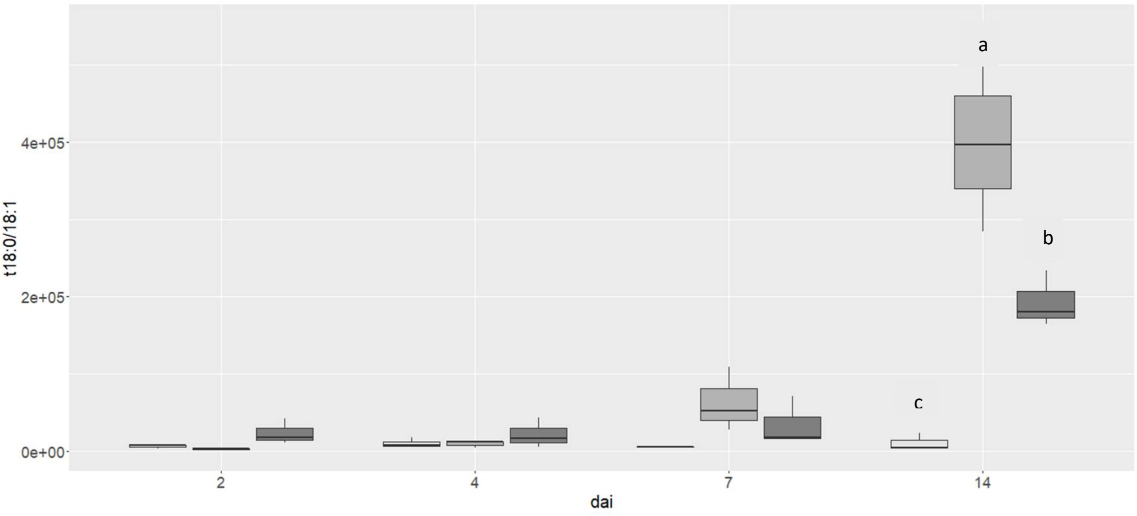
t18:0/18:1
4e+05
2e+05
0e+00
2
4
7
14
dai
a
b
c

g)

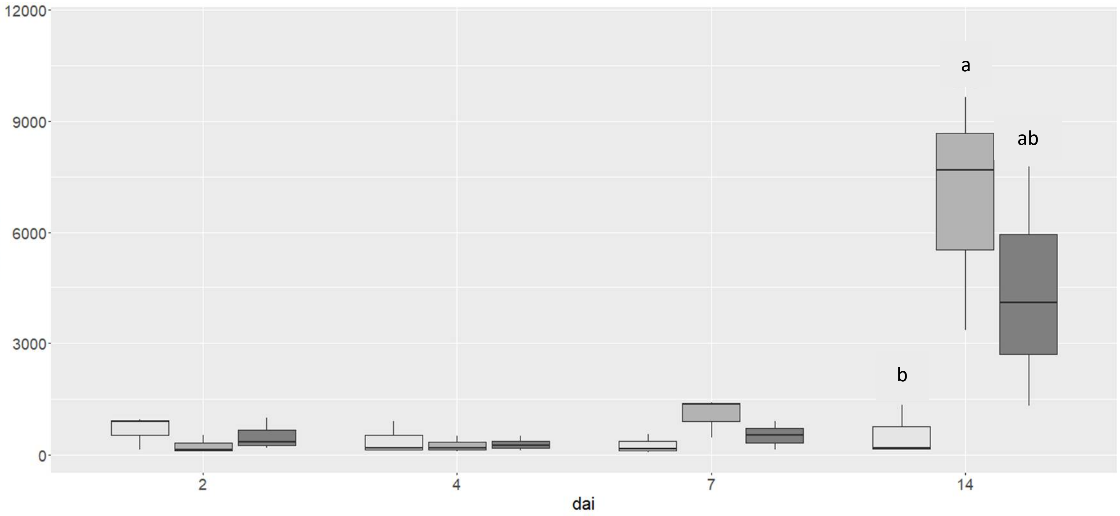
t18:1/16:0
12000
9000
6000
3000
0
2
4
7
14
dai
a
ab
b

h)

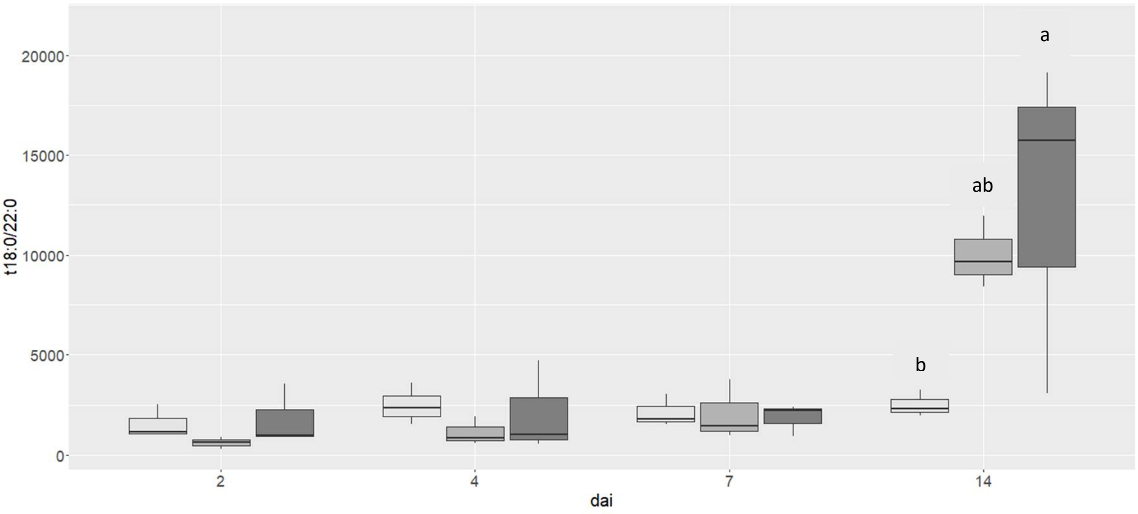
t18:0/22:0
20000
15000
10000
5000
0
2
4
7
14
dai
a
ab
b

i)

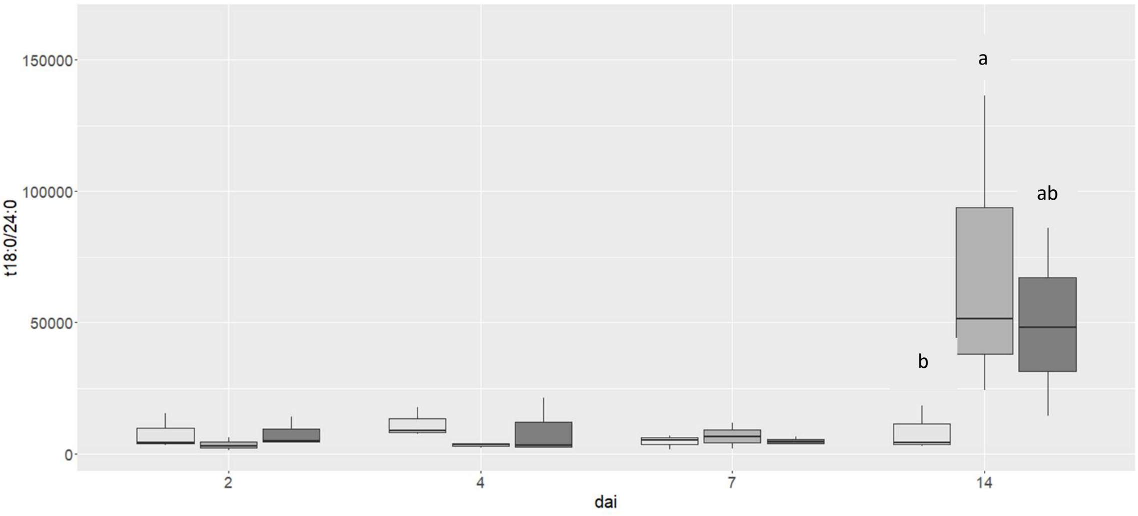
a
ab
b
150000
100000
50000
0
t18:0/24:0
2
4
7
14
dai

l)

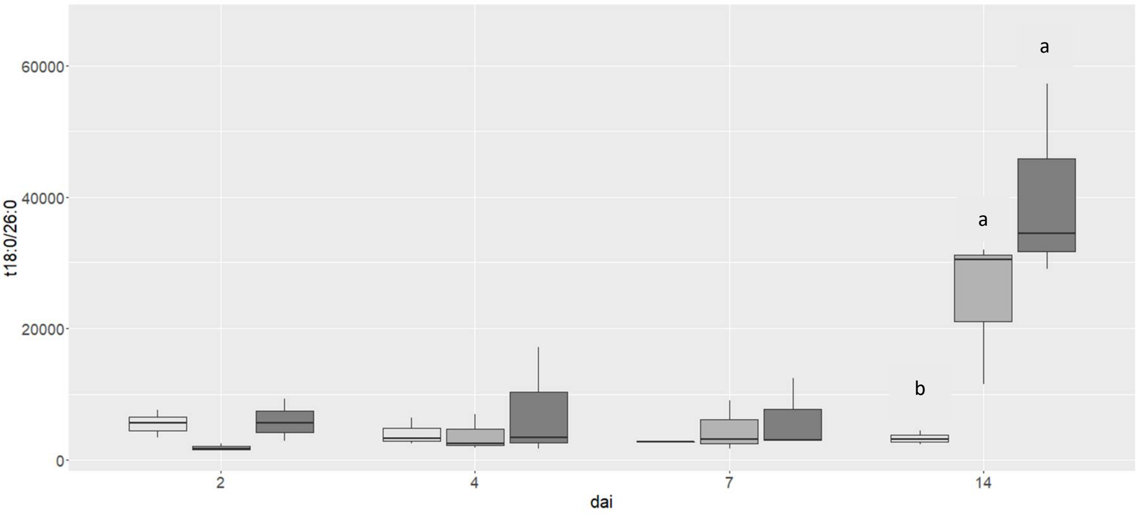
a
a
b
60000
40000
20000
0
t18:0/26:0
2
4
7
14
dai

m)

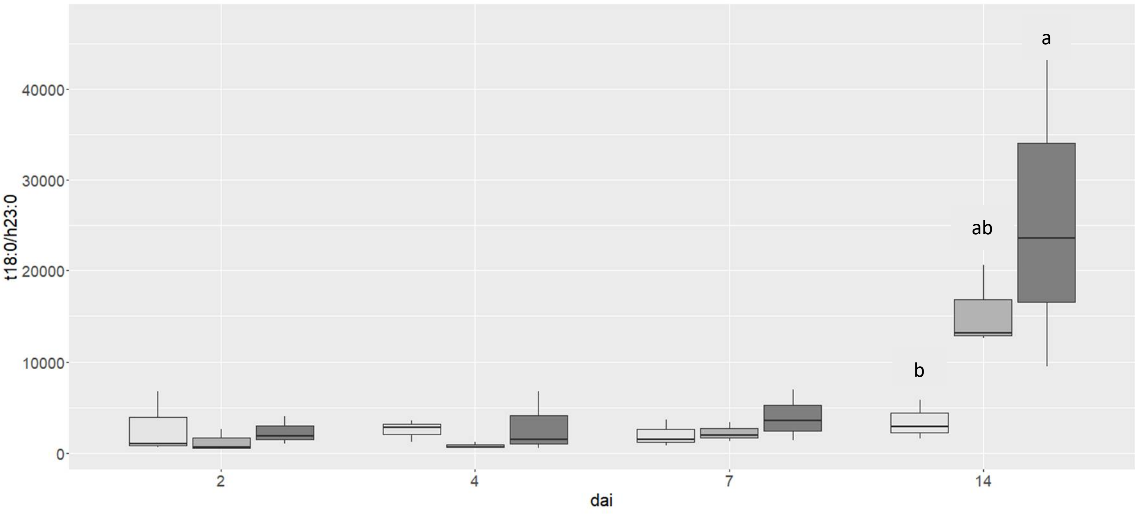
a
ab
b
40000
30000
20000
10000
0
t18:0/h23:0
2
4
7
14
dai

n)

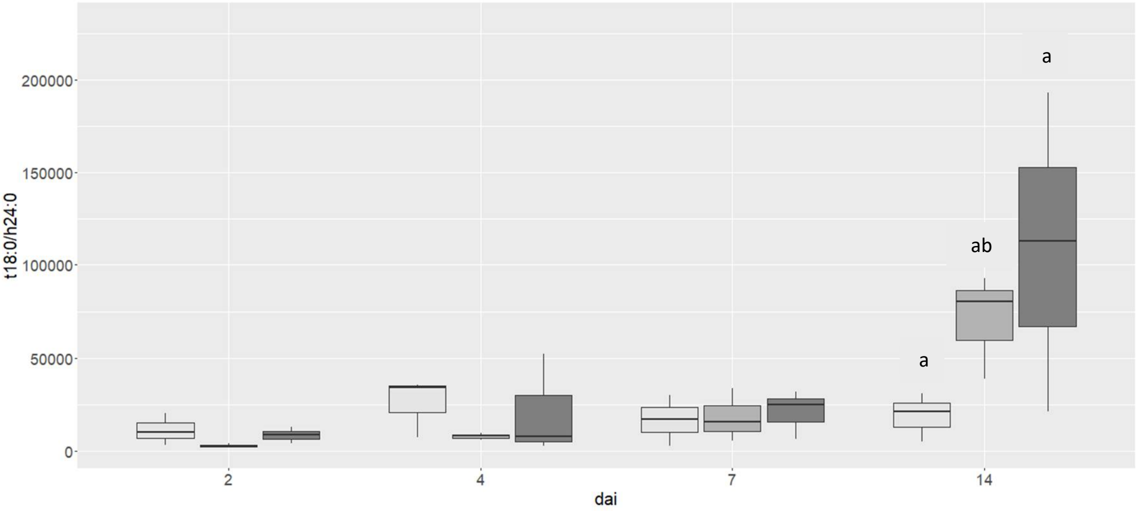

t18:0/h24:0
200000
150000
100000
50000
0
2
4
7
14
dai
a
ab
a

o)

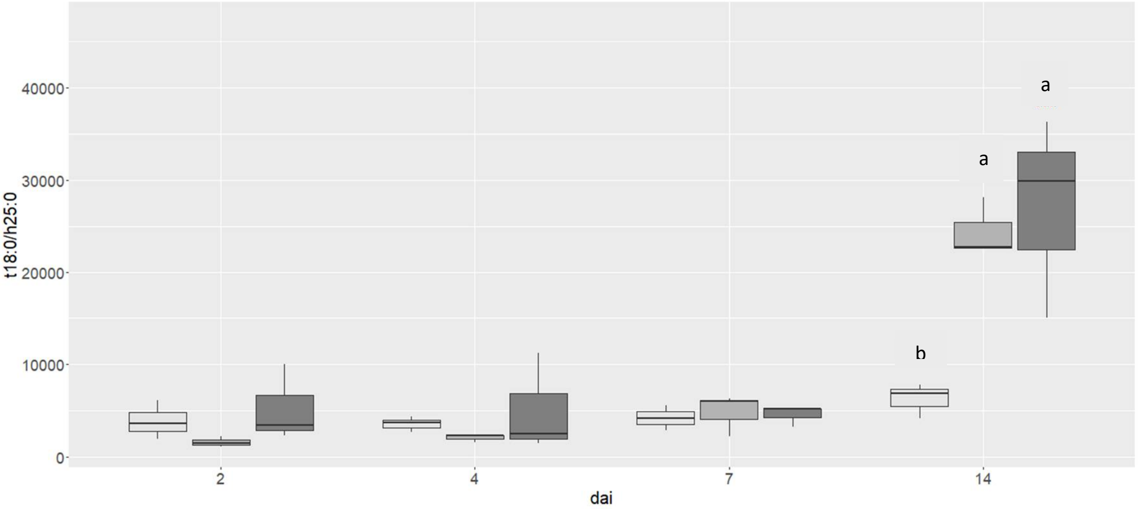

t18:0/h25:0
40000
30000
20000
10000
0
2
4
7
14
dai
a
a
b
